# ASO Author Reflections: How to Identify Patients at Genuinely High Risk of Recurrence from Localized Gastrointestinal Stromal Tumor of the Stomach?

**DOI:** 10.1245/s10434-021-09608-5

**Published:** 2021-03-09

**Authors:** Toto Hølmebakk, Kjetil Boye

**Affiliations:** 1 Department of Abdominal and Pediatric Surgery, Oslo University Hospital, The Norwegian Radium Hospital, Oslo, Norway; 2 Department of Oncology, Oslo University Hospital, The Norwegian Radium Hospital, Oslo, Norway; 3 Department of Tumor Biology, Institute for Cancer Research, Oslo University Hospital, The Norwegian Radium Hospital, Oslo, Norway

## Past

The overall survival benefit of adjuvant imatinib for localized GIST is uncertain,^1^,^2^ and clinicians generally maintain that the risk of recurrence should approach 50% for 3 years of treatment to be justified. For gastric GIST, the risk is low, and even in the high-risk category, recurrence-free survival exceeded 75% in one large study that administered adjuvant treatment only to a minority.^3^ Risk stratification is obscured by the increasing use of neoadjuvant treatment, which precludes assessment of mitotic activity. Other variables would be valuable, and there are indications that anatomical features are associated with outcomes.^4^ Population-based data specifically on patients with gastric GIST are lacking.

## Present

In this study, few recurrences were detected among patients with luminal or exophytic tumors, and without rupture, their prognosis was excellent irrespective of tumor size.^5^ These patients may not benefit from adjuvant imatinib. By contrast, a transmural growth pattern was a predictor of poor outcome and associated with increased mitotic activity. After neoadjuvant treatment, when the mitotic index is unknown, the growth pattern could serve as a surrogate variable, with continued imatinib treatment reserved for patients with transmural tumors. Tumor genotypes had a characteristic anatomical distribution: *platelet-derived growth factor receptor-α (PDGFRA)*-mutated tumors were situated in the lower end of the stomach and *KIT*-mutated tumors in the upper end, where tumors with deletions involving codons 557 and 558 in *KIT* exon 11 were concentrated in the upper third. The incidence of *KIT/PDGFRA* wild-type tumors was only 4%. More than 90% of the tumors in the upper third harbored mutations associated with imatinib sensitivity. For patients with tumors in this location, when facilities for molecular testing are unavailable, the authors suggest that neoadjuvant treatment could nevertheless be started and that even adjuvant treatment could be considered.

## Future

The authors have documented associations between anatomical, molecular, and clinical characteristics of gastric GIST. These findings could stimulate studies to identify the underlying biologic processes that explain the multifarious nature of this disease together with their potential as biomarkers for therapeutic decisions. In oncology, there is an innate drive to expand therapy in the hope of curing or deferring disease. Equally important should be the restriction of therapy when benefit is unlikely. For patients with gastric GIST, further efforts should be made to identify those who could be spared the side effects, costs, and inconvenience of extensive adjuvant treatment.
